# Distinctive response of CNS glial cells in oro-facial pain associated with injury, infection and inflammation

**DOI:** 10.1186/1744-8069-6-79

**Published:** 2010-11-10

**Authors:** SeungHwan Lee, Yuan Qing Zhao, Alfredo Ribeiro-da-Silva, Ji Zhang

**Affiliations:** 1 The Alan Edwards Centre for Research on Pain, McGill University, 740, Dr. Penfield Ave. Montreal, Quebec, H3A 2B2, Canada; 2 Faculty of Dentistry; McGill University, Quebec, Canada; 3 Department of Neurology and Neurosurgery, Faculty of Medicine, McGill University, Quebec, Canada; 4 Department of Pharmacology, Faculty of Medicine, McGill University, Quebec, Canada

## Abstract

Oro-facial pain following injury and infection is frequently observed in dental clinics. While neuropathic pain evoked by injury associated with nerve lesion has an involvement of glia/immune cells, inflammatory hyperalgesia has an exaggerated sensitization mediated by local and circulating immune mediators. To better understand the contribution of central nervous system (CNS) glial cells in these different pathological conditions, in this study we sought to characterize functional phenotypes of glial cells in response to trigeminal nerve injury (loose ligation of the mental branch), infection (subcutaneous injection of lipopolysaccharide-LPS) and to sterile inflammation (subcutaneous injection of complete Freund's adjuvant-CFA) on the lower lip. Each of the three insults triggered a specific pattern of mechanical allodynia. In parallel with changes in sensory response, CNS glial cells reacted distinctively to the challenges. Following ligation of the mental nerve, both microglia and astrocytes in the trigeminal nuclear complex were highly activated, more prominent in the principal sensory nucleus (Pr5) and subnucleus caudalis (Sp5C) area. Microglial response was initiated early (days 3-14), followed by delayed astrocytes activation (days 7-28). Although the temporal profile of microglial and astrocyte reaction corresponded respectively to the initiation and chronic stage of neuropathic pain, these activated glial cells exhibited a low profile of cytokine expression. Local injection of LPS in the lower lip skin also triggered a microglial reaction in the brain, which started in the circumventricular organs (CVOs) at 5 hours post-injection and diffused progressively into the brain parenchyma at 48 hours. This LPS-induced microglial reaction was accompanied by a robust induction of IκB-α mRNA and pro-inflammatory cytokines within the CVOs. However, LPS induced microglial activation did not specifically occur along the pain signaling pathway. In contrast, CFA injection led to minor microglial morphological changes and an induction of IκB-α mRNA in the CVO regions; a significant increase in IL-1β and IL-6 mRNA started only at 48 hours post-injection, when the induced pain-related behavior started to resolve. Our detailed analysis of CNS glial response clearly revealed that both nerve injury and oro-facial infection/inflammation induced CNS glial activation, but in a completely different pattern, which suggests a remarkable plasticity of glial cells in response to dynamic changes in their microenvironment and different potential involvement of this non-neuronal cell population in pathological pain development.

## Introduction

Oro-facial pain is frequently observed in dental clinics. One of the most common causes of neurogenic oro-facial pain is traumatic lesion of the trigeminal nerve [1]. Certain dental treatments, such as extraction of impacted third molars or extraction of endodontic material into the mandibular canal, are frequently implicated. Orthognathic surgery and surgery to the middle third of the face have also been implicated. Other events, such as stretching of the nerve, trapping of the nerve in scar tissue, or compression by inflammatory reactions, are also likely to be the causes. This type of hypersensitivity is severely debilitating, becomes chronic, can last for several months or years, even after the healing of the original trauma. Another oro-facial pain that has a well-understood etiology is that associated with infection. Infection or inflammation in teeth and periodontal tissues or in mucogingival tissues lead to inflammatory oro-facial pain. Pain associated with inflammation or infection declines in a predictable fashion as the infection recedes and there is tissue repair [2].

The sensory innervation of the oro-facial area is provided by branches of the trigeminal nerve. The great majority of trigeminal sensory neurons have their cell bodies clustered in the trigeminal ganglion [3]. Their central branches enter the brainstem at the level of the pons and end mostly in the principal sensory and spinal trigeminal nuclei [4]. Sensory information, including nociceptive information is conveyed via second-order neurons to higher centers [4]. It has been established for a long time that neurons are responsible not only for the transmission but also for the modulation of sensory information, including pain-related information. However, considerable data is emerging indicating that a variety of non-neuronal cells along the neuronal pain signaling pathways play an important modulatory role, both in the CNS and in the periphery [5]. Along these lines, cells of the immune system are recruited to the sites of peripheral injury and inflammation. Through a coordinated release of multiple classes of inflammatory mediators, these immune cells contribute to the activation and sensitization of the nociceptors in the primary afferents [6]. In the CNS, although they act as bystanders in nociceptive processes under normal conditions, both microglia and astrocytes become activated following damage to the peripheral or central nervous systems, release mediators to induce or/and maintain hyperexcitability in pain signaling and thereby contribute to abnormal nociception [7]. Despite that infection or inflammation can also stimulate the glia, the involvement of CNS glia in the modulation of inflammatory pain was not clearly identified.

Tissue macrophages are innate immune cells with well-established roles in the primary response to pathogens, and also in tissue homeostasis. Macrophage activation has a large spectrum of profiles, ranging from classically activated M1 macrophages, which have the capability to produce cytokines that enhance the immune response, to alternatively activated M2 macrophages, which possess important immuno-modulatory, tissue repair and remodeling properties [8]. Inflammatory pathogens favor macrophage differentiation toward an M1 profile, while tissue debris leads macrophage into regulatory and wound-healing M2 macrophages [9]. As resident macrophages in the CNS, microglia also have remarkable plasticity that allows them to efficiently respond to environmental signals and lead to distinct signaling cascade within the cells and different morphology changes or secretory activities [10]. There is no classification of activated microglia similar to that developed for activated macrophages. However, it is predictable that the response of microglia to infection, to remote damage on the peripheral tissue or nerve, and to direct lesion on the brain or spinal cord could be different, which would lead to different functional consequences.

To better understand the multi-dimensional profiles of glial activation in response to different insults and to clarify the potential contribution of glial cells in different types of pathological pain, in this study we compared the activation of microglia and astrocytes following nerve injury, infection and inflammation in the oro-facial region. Three animal models of pathological pain were used, including 1) neuropathic pain model following trigeminal nerve injury, 2) local infection-like pathology following subcutaneous injection of a bacterial toxin, lipopolysaccharide (LPS) and 3) local sterile inflammation following subcutaneous injection of complete Freund's adjuvant (CFA). Subsequently, we analyzed the phenotype and functional characteristics of glial cells in response to the above insults and correlated the observed changes with pain-related behavior.

## Materials and methods

### Animals

Adult male Sprague-Dawley rats (Charles River, Quebec, Canada) were used and weighed 250-275 g at time of surgery. Before surgery, they were acclimatized to standard laboratory conditions (14-h light, 10-h dark cycle) and given free access to rat chow and water. All protocols were performed in accordance with guidelines from the Canadian Council on Animal Care and the International Association for the Study of Pain, and were approved by the McGill University Animal Care Committee.

### Animal models

#### Mental nerve ligation

Rats were deeply anesthetized with isoflurane. As described previously [11], under standard surgical aseptic conditions, the mental nerve was exposed bilaterally starting at its exit point from the mental foramen. Approximately 6 mm of nerve was freed of adhering tissue and two silk ligatures (8.0) were tied loosely around it, as close as possible to the exit of the nerve from the bone, but with approximately 2 mm between ligatures. The tightness of the ligature was verified as the knots were just barely movable around the mental nerve. Sham-operated animals underwent the exact same surgical procedure with the exception of the nerve ligation. Lesions were performed bilaterally. Also, it has been reported that direct grooming behavior, indicative of pain, were more consistently observed following bilaterally lesion [12].

#### Subcutaneous injection of LPS

Under brief anesthesia with isoflurane, Lipopolysaccharide (LPS), from *Escherichia coli *strain 055:B5 (Sigma, L2880) was injected subcutaneously (1 mg/kg of body weight, dissolved in 50 μl saline) close to the midline of the lower lip. Dose was selected according to literature where systemic or local LPS induced a significant inflammatory reaction and adjusted in our pilot study for pain response.

#### Subcutaneous injection of CFA

the Complete Freund's Adjuvant (CFA) solution was prepared by thorough mixing 10 ml of Incomplete Freund's Adjuvant (IFA) (Difco laboratories) with 100 mg desiccated mycobacterium tuberculosis, strain H37Ra (Difco laboratories). Once prepared, it was kept at 4°C until use. Under brief anesthesia with isoflurane, a volume of 70 μl of CFA was injected subcutaneously in the middle part of the lower lip. Dose was established according to previous study in inducing inflammatory pain in paw and adjusted to the injection site (lower lip).

### Behavioral testing

Animals were habituated to the testing environment daily for at least two days before baseline testing. All animals were assessed for mechanical allodynia before surgery or injection and at specified time points after nerve injury, local LPS and CFA injection, respectively, until they were sacrificed for histological studies. Mechanical sensitivity was assessed using calibrated von Frey Hairs as described [13]. Animals were placed in boxes on an elevated metal mesh floor and allowed 40 to 60 min for habituation before testing. A series of von Frey filaments with logarithmically incrementing stiffness (Stoelting) was applied perpendicular to the mid-area of the lower lip. The 50% head escape threshold was determined using Dixon's up-down method as previously described [14].

### Tissue preparation

#### For histological studies

Rats were deeply anaesthetized with ketamine/xylazine and then perfused transcardially with 0.9% saline followed by 4% paraformaldehyde (PFA) in 0.1 M sodium phosphate buffer (pH 7.4). Brains were removed and placed in the same fixative overnight at 4°C then transferred to 30% sucrose in phosphate buffer at 4°C for cryoprotection for at least 18 hours. Brains were cut transversely into 30-μm-thick sections on a sliding freezing microtome, collected in an anti-freeze solution [0.05M sodium phosphate buffer (pH 7.3) containing 30% ethylene glycol and 20% glycerol] and stored at -20°C until use.

#### For real time PCR experiments

Rats were deeply anaesthetized with isoflurane and decapitated, then segments of brainstem, including the Sp5C and AP areas were quickly removed and snap frozen in liquid nitrogen and stored at - 80°C until use.

### Immunohistochemistry (IHC)

A standard bright field immunohistochemistry protocol was applied to characterize the spinal glial cell reaction to peripheral nerve injury, to LPS and to CFA injection. Free-floating sections were first extensively washed in phosphate buffer and then incubated overnight at 4°C with the following antibodies, diluted in phosphate buffer containing 0.25% triton-X and 0.1% normal goat serum: rabbit anti-ionized calcium-binding adaptor molecule 1 (Iba-1) polyclonal antibody (for microglia and macrophages; 1:1000; Wako Chemicals, Richmond, VA) and rabbit anti-glial fibrillary acid protein (GFAP) polyclonal antibody (for astrocytes; 1:1000; DakoCytomation, Carpinteria, CA) and, after washing in phosphate buffer then incubated with a biotinylated secondary antibody (1:1000, Vector Laboratories, Canada), followed by an avidin-biotin-peroxidase complex (Vectostain ABC Elite Kit, Vector Laboratories). After several washes in phosphate buffer, tissue sections were reacted in 0.05% diaminobenzidine and 0.003% hydrogen peroxide. Sections were then mounted on Super Frost plus slides, counterstained with thionin to facilitate the visualization of anatomical boundaries, dehydrated through graded concentrations of ethanol and coverslipped with DPX.

To identify the phenotype of IL-1β expressing cells in LPS-treated rats, immunofluoresecent staining with antibodies against IL-1β (1:500, Santa Cruz) and OX-42 (1:1000, Cedarlane) was performed in brain CVO regions.

### *In situ *Hybridization (ISH)

Detection of mRNAs encoding IκB-α and IL-1β was performed on brain sections using 35S-labeled riboprobes. cDNA fragments containing 1114 bp (Genebank U36277) and 700 bp (Genebank NM_031512) were used as probes for IκB-α and IL-1β, respectively. Riboprobe synthesis and hybridization were performed as per a previously described protocol [15]. Briefly, plasmids were linearized, sense and anti-sense cRNA probes were synthesized with appropriate RNA polymerase. Sections were postfixed in 4% PFA and digested by proteinase K (10 μg/ml, at 37°C for 20 min). After which brain sections were rinsed in water and by a solution of 0.1 M triethanolamine (TEA, pH 8.0), acetylated in 0.25% acetic anhydride in 0.1 M TEA and then dehydrated. Hybridization of the sections by riboprobe involved a 10^6 ^cpm/ml/slide of hybridization mixture and incubation at 55°C overnight in a slide warmer. Slides were then rinsed in standard saline citrate buffer (1X SSC: 0.15 M NaCl, 15 mM trisodium citrate buffer, pH 7.0) and digested by RNase A at 37°C (20 μg/ml), rinsed again in descending concentrations of SSC, and dehydrated through graded concentrations of ethanol. Sections were exposed to x-ray film (BioMax, Kodak, Rochester, NY) for 2-4 days and dipped in NTB2 nuclear emulsion (diluted 1:1 with distilled water, Kodak). Slides were kept at 4°C for 2-4 weeks safe from light and developed in D19 developer (Kodak).

### RNA extraction and real-time quantitative PCR

Total RNA was extracted from segments of brainstems, including the Sp5C and AP areas using the Rneasy Lipid Tissue Mini Kit (QIAGEN). Synthesis of cDNA from total RNA was performed with the SuperScript VILO cDNA Synthesis Kit (Invitrogen). Primers were produced by QIAGEN QuantiTect (IL-1β-QT00181657, IL6-QT00182896, GAPDH-QT00199633). Fifteen samples were analysed (Naïve; nerve injured-day 7; local LPS-injected-5 hours; local CFA- injected-5 hours; local CFA-48 hours; n = 3/group). Experiments were performed in triplicate using an applied Biosystems 7900HT Fast Real-Time PCR System. Levels of target mRNAs were normalized to the housekeeping gene GAPDH. Fold changes versus naïve animals were analyzed using the comparative Ct (dCT) method [16].

### Image analysis and cell counting

Images were captured using Olympus BX51 microscope (Tokyo, Japan) equipped with a colour digital camera (Olympus DP71). Cell counting of both Iba-1 and GFAP positive cells was performed on digitized images. Defined areas of interest (AOIs) on Sp5C and Pr5 were selected. Only a well-defined thionine-stained nucleus associated with a well-circumscribed, immunopositive cell body was considered as a particular phenotype, e.g., Iba-1+ microglia and GFAP+ astrocytes. The cell counting was conducted on 3-4 sections per animal and five animals in nerve injured group and three animals in sham-operated group.

### Statistical analysis

**Figure 1:**
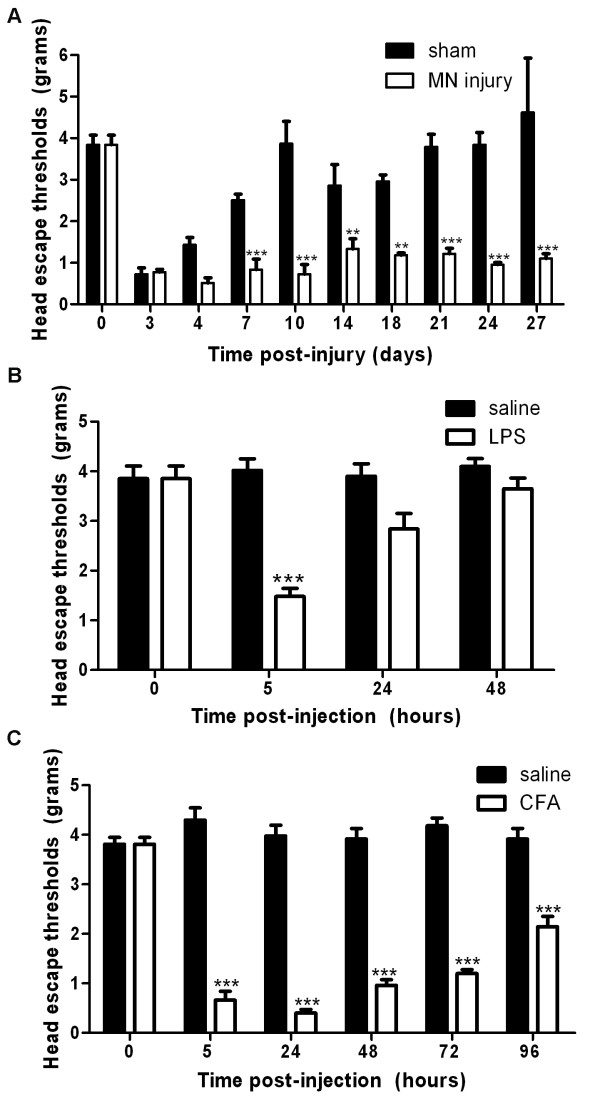
**Trigeminal nerve injury, local infection and inflammation induced mechanical allodynia of the lower lip area**. **A)**. In rats having mental nerve (MN) ligation (n = 5), head escape threshold to von Frey stimulation decreased shortly after the injury (day 3) and remained low until the end of experiment (day 27). The decrease threshold observed in sham-operated rats (n = 3) shortly post-surgery disappeared completely after 10 days. **B)**. Subcutaneous injection of LPS induced an acute mechanical hypersensitivity of the lower lip, which was significant at 5 hours post-injection (n = 5/group). **C)**. CFA injection (n = 3 saline, n = 5 CFA) triggered mechanical allodynia during first three days post-injection and then progressively disappeared. ** P < 0.01, *** P < 0.001, compared to sham-operated group at their corresponding time point.

**Figure 2:**
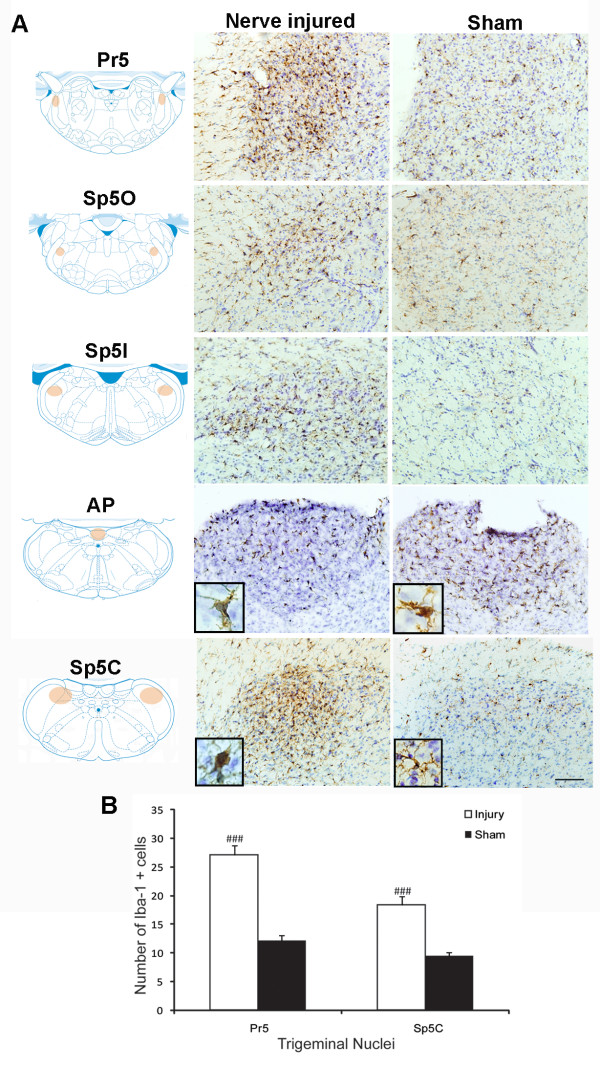
**Microglial reaction in response to mental nerve ligation at 14 days post-injury**. **A)**. Immunostaining for Iba-1 in four trigeminal subnuclei, including principal sensory nucleus (Pr5), spinal oralis (Sp5O), spinal interpolaris (Sp5I) and spinal caudalis (Sp5C). A significant increase in Iba-1 immunoreactivity was observed in nerve injured rats, particularly in Pr5 and Sp5C, together with remarkable changes in microglial cell shape (inserts). Note there are no significant changes of Iba-1 staining and microglial cells shape in medullar CVOs, area postrema (AP). Diagrams were adapted and modified from [59]. Orange shadowed area indicated where the pictures were taken. Scale bar: 50 μm **B)**. The density of Iba-1+ cells in Pr5 and Sp5C areas was significant higher in injured group than that of sham-operated. Data are expressed as Mean ± SEM. ### P < 0.001, compared to sham-operated group.

**Figure 3:**
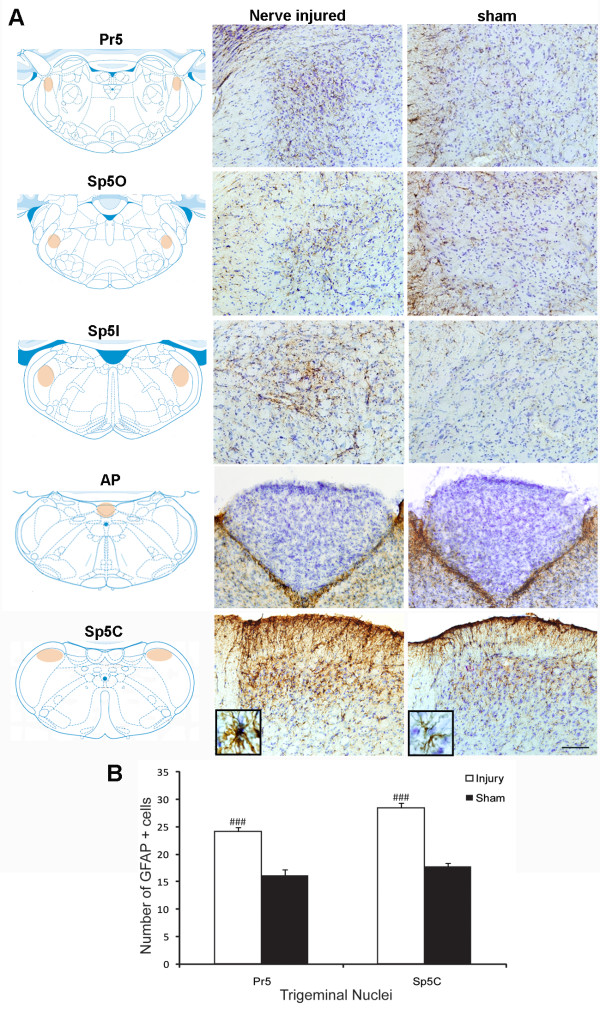
**Astrocyte reaction in response to mental nerve ligation at 14 days post-injury**. **A)**. Immunostaining for GFAP in four trigeminal subnuclei, including the principal sensory nucleus (Pr5), spinal oralis (Sp5O), spinal interpolaris (Sp5I) and spinal caudalis (Sp5C). There was a significant increase in GFAP immunoreactivity in nerve injured rats, which was more prominent in Pr5 and Sp5C, together with remarkable changes in astrocyte cell shape (inserts). Note there are no significant changes of GFAP staining in medullar CVOs, area postrema (AP). Diagrams were adapted and modified from [59]. Orange shadowed area indicated where the pictures were taken. Scale bar: 50 μm. **B)**. The density of GFAP+ cells in Pr5 and Sp5C areas was significant higher in injured group than that of sham-operated. Data are expressed as Mean ± SEM. ### P < 0.001, compared to sham-operated group.

All data are presented as means ± SEM. Statistic significance was determined using: 1) Two way ANOVA analysis for behavioural data in Figure 1; 2) unpaired t-test for the difference of cell numbers between injured- and sham-operated group in Figure 2 and Figure 3; and unpaired t-test for the difference of cytokine expression in each challenged group vs. naive in Figure 10. The criterion for statistical significance was p < 0.05.

## Results

### Nerve injury, infection and inflammation induced mechanical hypersensitivity

After bilateral mental nerve ligation, rats developed mechanical allodynia, as tested in the area of the lower lip close to the midline (Figure 1A). From post-injury day 3 onwards, the head escape threshold elicited by the calibrated Von Frey hair was significant lower than the baseline before surgery; between 0.51 ± 0.13 g to 0.78 ± 0.06 g during the first 10 days, and then remained stable around 1.0 g to the end of testing period (day 27); in contrast, the threshold before surgery was 3.84 ± 0.186 g. The hypersensitivity observed up to 10 days may be in part caused by the incisions which are very close to the testing area. In agreement with this, sham-operated animals also manifested a mechanical hypersensitivity which was significant at 3 days post-surgery and recovered after 10 days.

Rats receiving subcutaneous injection of LPS in the lower lip skin developed an acute mechanical allodynia (Figure 1B) which was significant at 5 hours post-injection (1.48 ± 0.17 g) and disappeared progressively afterwards (2.84 ± 0.31 g at 24 hrs and 3.65 ± 0.22 g at 48 hrs); baseline value was 3.79 ± 0.47 g. The head escape threshold in CFA injected rats started to decrease 5 hours post-injection (0.66 ± 0.18 g), reached its lowest level at 24 hours (0.4 ± 0.07 g) and started to recover after 48 hours (Figure 1C).

### Peripheral nerve injury triggered glial activation in the brain

**Figure 4:**
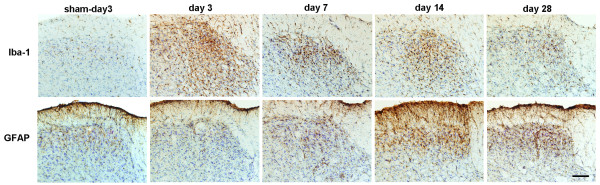
**Temporal profile of microglial and astrocyte activation following ligation of the mental nerve**. Microglia and astrocytes were immunolabeled with Iba-1 and GFAP, respectively. The intensity of Iba-1 immunoreactivity (ir) in Sp5C area was increased between days 3-days 14 and sharply diminished after days 28 post-injury whereas GFAP immunoreactivity increased significantly after days 14 and remained elevated at least until days 28. Scale bar: 50 μm.

Ligation of mental nerve did trigger a reaction of both microglia and astrocytes within the trigeminal complex in the brainstem area. The anatomical distribution (both spatial and temporal) and morphological changes of activated microglia and astrocytes were visualized using Iba-1 and GFAP, respectively, as cellular markers. Iba-1 labeled microglia were distributed almost homogenously throughout the entire brainstem in sham-operated rats. In nerve injured animals, Iba-1 staining was significantly increased in the trigeminal nuclear complex, especially in the principal sensory nucleus (Pr5) and subnucleus caudalis (Sp5C) area (Figure 2). Activated microglia in Pr5 and in Sp5C were characterized by a dramatically enlarged soma, short/thick and fewer ramifications than resting microglia (Figure 2A, inserts). A significant glial cell proliferation was observed 14 days after nerve ligation. The number of Iba-1^+ ^cells in Pr5 and Sp5C regions (27.18 ± 1.64, 18.3 ± 1.6 respectively) in nerve injured rats was 2-3 times higher than (12.23 ± 0.86 in Pr5 and 9.54 ± 0.57 in Sp5C) in sham-operated rats (Figure 2B). GFAP stained astrocytes displayed signs of activation in the trigeminal nuclei (Pr5 and Sp5C) (Figure 3) having major hypertrophic changes in morphology, with large, thick branches (Figure 3A, inserts) and a relatively slight increase in cell density (24.22 ± 0.74-injured vs 16.12 ± 1.04-sham in Pr5 and 28.51 ± 0.82-injured vs. 17.74 ± 0.71-sham in Sp5C) (Figure 3B) The temporal profile of microglial and astrocyte activation in trigeminal nuclei following mental nerve injury was similar to what we have previously reported spinal glial activation following sciatic nerve injury [17]. Microglial response was initiated early (day 3-day 14), followed by a delayed astrocyte activation (d7-d28) (Figure 4). No significant changes in microglia or astrocytes were detected in other brain regions, including those related to the pain perception and modulation, such as the thalamus and somatosensory cortex (data not shown).

### Oro-facial infection-triggered glial reaction in the brain

**Figure 5:**
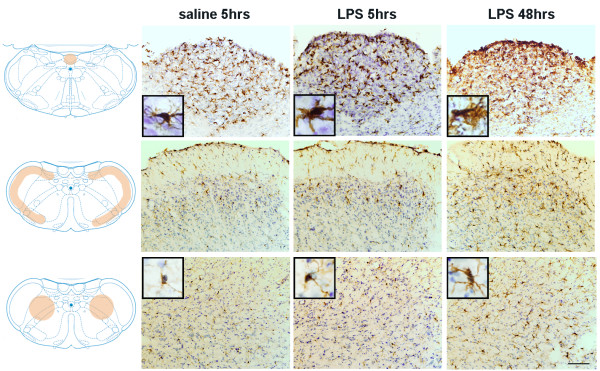
**Subcutaneous injection of LPS in the lower lip induced microglial activation in the brain**. In response to local LPS administration, microglial activation was first observed in the CVOs, such as the area postrema (AP) and at the edge of the grey matter at 5 hours post-injection. At 48 hours, activated microglia were dispersed throughout the parenchyma. Activated microglia exhibited remarkable changes in microglial morphology (inserts). Diagrams were adapted and modified from [59]. Orange shadowed area indicated where the pictures were taken. Scale bar: 50 μm.

To analyze the glial response to local oro-facial infection/inflammation, in addition to the trigeminal complex in the brainstem, we have paid special attention to regions where the capillaries in these structures are fenestrated and the blood brain barrier (BBB) is not complete, including circumventricular organs (CVOs), ventricles and choroid plexus. Five hours following LPS injection, at a time where an acute mechanical allodynia was detected in the lower lip, we found some morphological changes of Iba-1^+ ^microglia in the CVOs, such as area postrema (AP) where the soma of these Iba-1^+ ^cells started to enlarge, and displayed thick and rigid processes (Figure 5, mid-column). Some enlarged Iba-1^+ ^cells also appeared at the edge of the grey matter (Figure 5, mid-column), but we did not observe any evidence of microglial activation restricted to the trigeminal complex. However, 48 hours later, when the mechanical hypersensitivity in the lower lips was completely resolved, scattered, enlarged, Iba-1 intensely labeled microglia were found across the entire parenchyma (Figure 5, right-column). No significant changes on GFAP labeled astrocytes were detected at any time, in any regions of the brain (data not shown).

### Oro-facial sterile inflammation-triggered glial reaction in the brain

**Figure 6:**
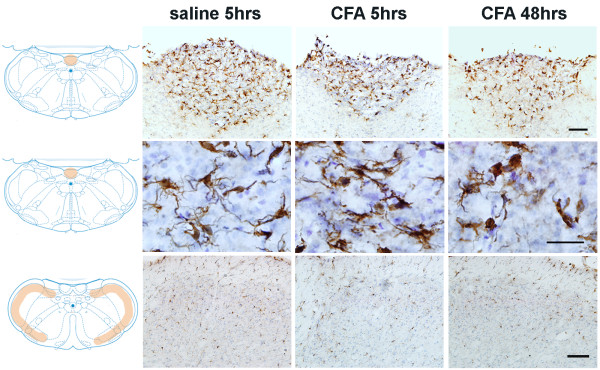
**Subcutaneous injection of CFA in the lower lip induced microglial activation in the brain**. In response to local CFA, microglial activation was only observed in the CVOs, such as the area postrema (AP) (upper row), with minor changes in microglial morphology at 5 and 48 hours post-injection (middle row). No activated microglia were found at the edge of the grey matter. Diagrams were adapted and modified from [59]. Scale bar: 50 μm.

Compared with LPS induced CNS glial response, local CFA injection only triggered a minor morphological change on Iba-1^+ ^microglial cells in CVOs (Figure 6). No significant changes, including intensity of Iba-1 labeling and iba-1 labeled cell shape were detected in the brain parenchyma (Figure 6). No significant changes on GFAP labeled astrocytes were detected at any time, in any regions of the brain (data not shown).

### Activation of NF-κB and expression of proinflammatory cytokines following nerve injury, infection and sterile inflammation in oro-facial area

Secretion of proinflammatory cytokines is one of the key elements of glial activation. Many *in vitro *and *in vivo *data suggested that proinflammatory cytokines, together with other inflammatory mediators can directly excite neurons and act as signaling molecules between activated glial cells and neurons. To further understand the functional aspects of glial activation in response to different pain-related insults, in addition to anatomical distribution and morphological changes, we also examined the activity of NF-κB (a transcription factor responsible for the activation of many inflammation related genes) and the capability of activated glial cells to produce proinflammatory cytokines.

**Figure 7:**
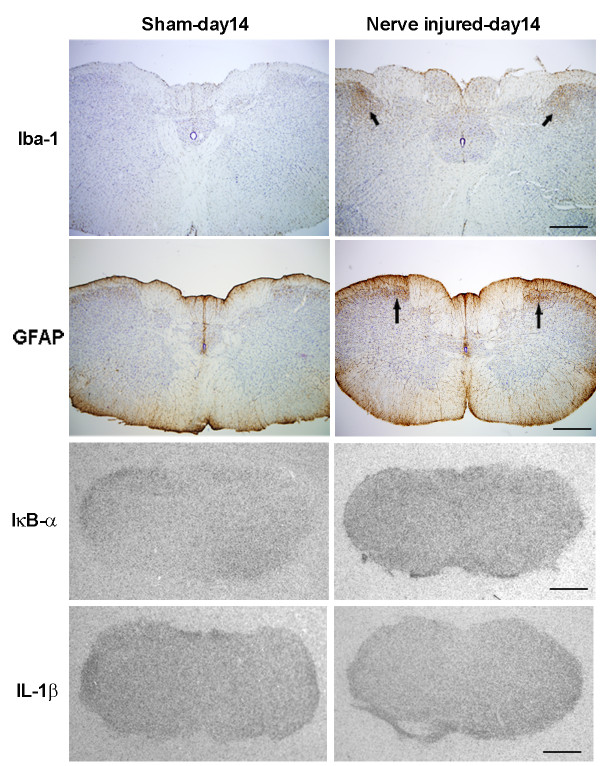
**Detection of IκB-α and IL-1β mRNAs on activated glial cells in Sp5C with *in situ *hybridization**. Although increases in Iba-1 and GFAP immunoreactivities were found in Sp5C areas 14 days after the ligation on the mental nerve (arrows), no positive hybridization signals for IκB-α or IL-1β mRNAs were detected in these regions (lower panels). Scale bar: 500 μm.

**Figure 8:**
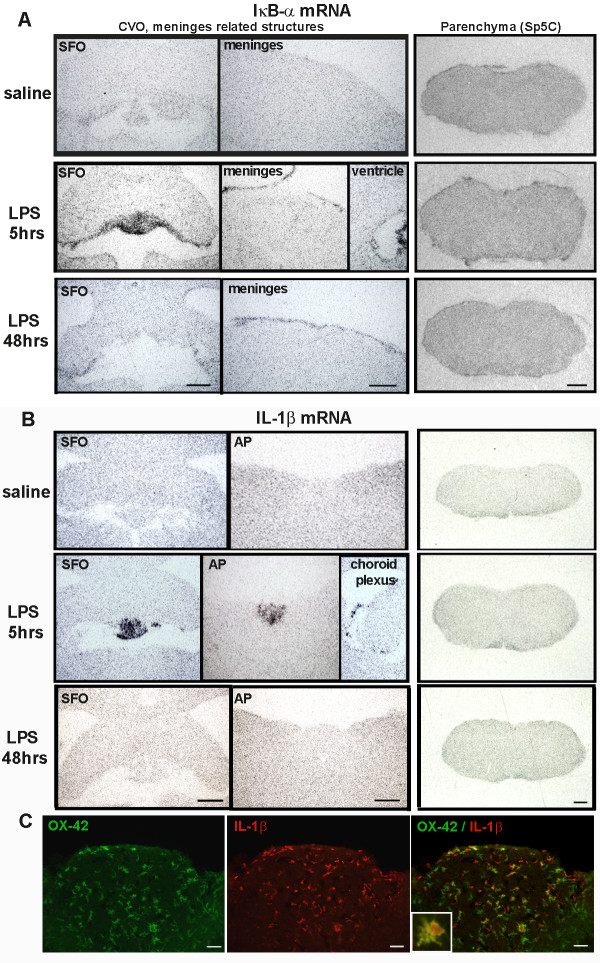
***In situ *hybridization detected robust expression of IκB-α and IL-1β mRNAs in the brain following local LPS injection**. **A)**. IκB-α mRNA was dramatically increased in the CVOs, such as SFO, meninges and ventricle related structures at 5 hours post LPS injection, and this increase was significantly reduced at 48 hours. No positive signals were detected within the parenchyma, including Sp5C. **B)**. IL-1β mRNA was also induced in the CVOs, such as SFO, AP and choroid plexus at 5 hours post LPS injection, it was almost completely disappeared at 48 hours. No positive signals were detected within the parenchyma, including Sp5C. **C)**. Double immunofluorescent labelling demonstrated that some of IL-1β^+ ^cells are OX-42^+ ^microglia. Scale bar: 500 μm in A and B; 50 μm in C.

**Figure 9:**
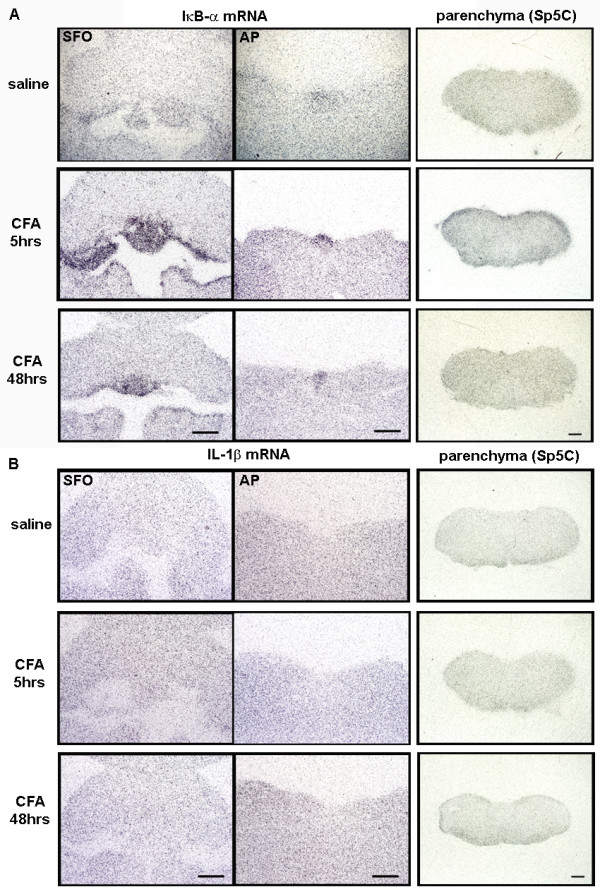
***In situ *hybridization detected robust expression of IκB-α mRNA in the brain following local CFA injection**. **A)**. IκB-α mRNA was dramatically increased in the CVOs, such as SFO and AP at 5 hours post CFA injection, and this increase was reduced at 48 hours. No positive signals were detected within the parenchyma, including the Sp5C. **B)**. No positive *in situ *hybridization signals for IL-1β mRNA were detected at any examined time points (5 and 48 hours) after CFA injection, in any region of the brain. Scale bar: 500 μm.

For this, we used initially *in situ *hybridization approaches to investigate mRNA expression of inhibitor of NF-κB (IκB-α), an index for NF-κB activity, and cytokine IL-1β expression in the brains of three different models. Surprisingly, although both microglia and astrocytes were highly activated in the Sp5C area, we were not able to detect the mRNA expression for IκB-α and IL-1β in the corresponding regions from day 3 to day 14 post-injury. Figure 7 illustrates the microglial and astrocyte activation evidenced by Iba-1 and GFAP staining (arrows) in Sp5C regions and lack of IκB-α and IL-1β mRNA positive signals with ISH detection at 14 days following mental nerve ligation. However, in local LPS-treated animals, at 5 hours post-injection both IκB-α (Figure 8A) and IL-1β (Figure 8B) mRNAs were highly expressed and restricted to the CVOs, ventricles and choroid plexus; some of these IL-1β expressing cells in CVOs were OX-42 positive microglia (Figure 8C). The positive hybridization signals were significantly reduced at 48 hours. Either at 5 hours, when animals showed an acute painful response or at 48 hours, when Iba-1 labeled enlarged microglia were found in the brain parenchyma, none of IκB-α and IL-1β expression was detected with *in situ *hybridization in the pain transmission-related structures, such as the trigeminal nuclei (Figure 8). In CFA treated rats, only IκB-α mRNA was detected in the CVOs (Figure 9).

**Figure 10:**
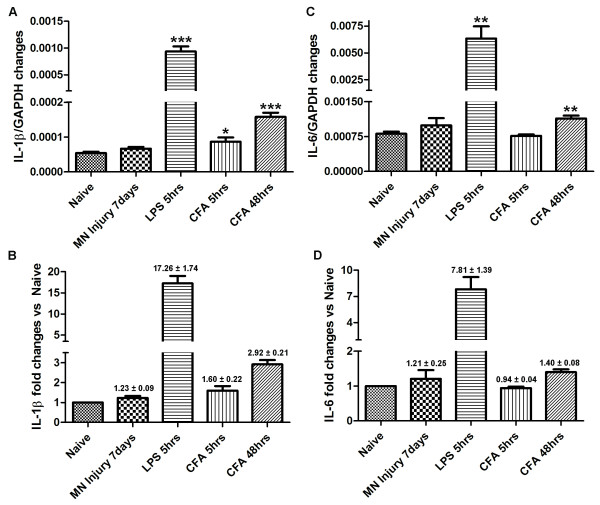
**Expression of proinflammatory cytokines IL-1β and IL-6 in the brainstem measured by quantitative real time PCR**. **A)**. IL-1β/GAPDH mRNA expression in each group. **B)**. Fold changes in IL-1β expression (challenged rats vs. naïve rats). **C)**. IL-6/GAPDH mRNA expression in each group. **D)**. Fold changes in IL-6 expression (challenged rats vs. naïve rats). Data are expressed as Mean ± SEM. * P < 0.05, ** P < 0.01 compared to naïve rats. N = 3/group.

Having concerns on the sensitivity of the *in situ *hybridization approach, we decided to examine the expression of these inflammatory molecules (IL-1β and IL-6) with real-time PCR. Segments of brainstem, including the Sp5C and AP areas, were collected from rats having nerve injury (7 days), LPS injection (5 hours), and CFA injection (5 and 48 hours). Results yielded from PCR amplification demonstrated that basal expression of IL-1β (6.7 ± 0.05 × 10^-5 ^fold changes vs. GAPDH) and IL-6 (9.92 ± 0.15 × 10^-4 ^fold changes vs. GAPDH) in naïve brain was extremely low (Figure 10A, C). Compared with naïve animals, mental nerve ligation did increase the expression of both IL-1β and IL-6 mRNAs in the brainstem, but only very slightly, as we detected a 1.23 ± 0.09 fold increases for IL-1β and a 1.21 ± 0.25 fold increases for IL-6 (Figure 10B, D), values which explain why we were not able to detect the signals with *in situ *hybridization. In contrast, subcutaneous injection of LPS in the lower lip induced a dramatic increase of both IL-1β (17.26 ± 1.74 fold increases vs. naive) and IL-6 (7.81 ± 1.39 fold increases vs. naive) in the brain (Figure 10B, D). CFA injection-stimulated cytokine expression in the brain significantly increased only after 48 hours, with a 2.92 ± 0.21 fold increases for IL-1β and 1.40 ± 0.08 fold increases for IL-6 (Figure 10B, D).

## Discussion

In this study, we characterized the morphological and functional profile of CNS glial cells in response to peripheral nerve injury, to local oro-facial infection and to inflammation. All these three insults triggered a specific pattern of mechanical allodynia on the lower lip. Our results demonstrated that 1) Mental nerve injury triggered both microglia and astrocyte activation in the region of the central terminals of the damaged nerve fibres. However these activated glial cells exhibited low capacity to produce proinflammatory cytokines. The spatial and temporal profile of this glial activation was closely correlated with the development of mechanical allodynia following nerve ligation. 2) LPS injection, a model of oro-facial infection, triggered an acute microglial reaction, starting from the CVOs, which diffused progressively into the CNS parenchyma; and was characterized by a robust induction of IκB-α and IL-1β, IL-6 mRNAs in the CVOs. Most likely this is an acute CNS inflammatory response, not directly relevant to the local mechanical hypersensitivity following LPS injection. No significant astrocyte activation was observed. 3) CFA injection, a model of oro-facial sterile inflammation, only triggered minor changes on microglial morphology and a transient induction of IκB-α mRNA, restricted in the CVOs, and a slight increase of IL-1β and IL-6 mRNAs at late time point, which is not likely correlated to the pain development. Therefore we conclude that nerve injury-induced microglial activation is characterized by morphological changes with a low profile of cytokine expression, whereas infection-induced microglial activation is accompanied by high levels of cytokine production. Astrocyte response was significant only following nerve injury.

### Three animal models used in this study provided diversified stimuli to examine the plasticity of CNS glial cells and their potential contribution in the development of pain

Lesion on the peripheral nerve is a major cause for neuropathic pain. Many functional and phenotypic changes occur along pain conducting pathways, from peripheral terminals to higher centers in the brain. In addition, as most animal models of neuropathic pain entail lesions of mixed nerves such as the sciatic nerve, which possesses sensory, motor and sympathetic fibers, the model that we used in this study, loose ligation on the mental nerve offered a unique opportunity to study the effects of lesion on sensory fibers, since in rats the mental nerve is almost exclusively sensory in origin [18,19]. As a component of the cell wall of Gram-negative bacteria, lipopolysaccharide endotoxin (LPS) is a well established agent to mimic the endogenous effect during infection and sepsis. Systemic injection of LPS induces fever and septic shock and is associated with an increase of different cytokines, including TNF-α, IL-1β and IL-6, in the blood circulation [20,21] and in the brain [22,23] in a pattern similar to that seen in natural infection. However, the impact of a local, peripheral injection of LPS on the CNS and relationship with local LPS induced pain behavior were not yet clarified. CFA is an immune response enhancer, effective in stimulating cell-mediated immunity and may lead to increase of the production of certain immunoglobulin in the absence of an infection. Due to its painful reaction and its immunopotentiator properties, CFA is frequently used to study sterile inflammatory reaction-associated pain, including arthritis [24,25]. In this study, we used CFA as a model of a sterile inflammatory condition.

### Nerve injury and infection/inflammation signal the CNS glial cells through different pathways

Within the central nervous system, microglia and astrocytes represent two highly reactive intra-parenchymal cell populations. Microglia, which originate from the bone marrow, provide immune surveillance of any exogenous or endogenous perturbation [26]. Activated microglia are characterized by a specific morphology, proliferation, increased expression of cell surface markers or receptors, and changes in functional activities such as migration to areas of damage, phagocytosis, and production/release of proinflammatory substances [27]. Developmentally different from microglia, astrocytes, normally support neurons by maintaining metabolic homeostasis, and also respond to different insults. The prominent morphological feature of astrocyte activation is hypertrophy. Functionally, this activation is manifested by increased production of a variety of trophic factors and by an alternation of astrocyte function in the maintenance of homeostasis. The fact that peripheral nerve injury can induce microglial/astrocyte activation has been demonstrated in several chronic neuropathic pain models [28-30]. Our detailed systematic analysis of mental nerve injury induced microglia and astrocyte activation in the subnucleus of trigeminal complex revealed a similar pattern of glial activation to that previously described in the spinal cord following sciatic nerve injury [17]. CNS glial response following peripheral nerve damage occurs through a neural pathway, at the region where the central terminals of the damaged nerve fibers end. In the current study, we observed that mental nerve ligation evoked microglia and astrocytes activation predominated at Sp5C where nociceptive primary afferents terminate, and at Pr5 area where larger A-beta afferent fibers synapse. Microglial response preceded that of astrocytes and astrocyte activation outlasted transient microglial activation. While dramatic morphological changes were observed in both microglial and astrocyte responses, cell proliferation also occurred, but the increase in cell numbers was higher for the microglial cell population. We did not find evidence of any significant changes in other pain related regions, either through direct projection from the trigeminal brain stem complex, or through multisynaptic pathways using relays in the reticular formation and adjacent brainstem areas, including thalamus, superior colliculus, periaqueductal gray and others (data not shown), although some previous studies observed glial activation in pain modulatory circuitry, such as rostral ventromedial medullar in a similar nerve injury model [31]. In contrast, inflammatory signals triggered in the periphery following either LPS or CFA injection likely reach the CNS via an endocrine-like mechanism. Indeed, in our study, the subcutaneous injection of LPS triggered an acute brain microglial response, including both morphological changes and a robust induction of cytokine expression, which was initiated from the CVOs, membrane lining on the ventricles and choroid plexus. What is common to these regions is that they are devoid of blood-brain barrier, therefore blood borne molecules have easier access to the CNS than in other regions. This is in agreement with previous observations that the direct action of LPS on cells bearing LPS receptor-CD14 within these organs initiates a rapid activation of myeloid derived cells, including microglia and perivascular macrophages [32,33]. Cytokines and other inflammatory molecules released by these activated microglia/macrophages spread the signals into the adjacent parenchyma in a migratory-like pattern [22]. We show that CFA seems capable of stimulating the glial reaction in the brain, but the pattern was similar to that following LPS.

### Characteristics of nerve injury and infection/inflammation-induced microglial activation

Microglia share the same origin with circulating monocytes and peripheral tissue macrophages and indeed represent CNS tissue specific macrophages. Therefore, it is not surprising that there could be a wide range of phenotypic and functional similarities between microglia cells and peripheral macrophages. Mainly based on *in vitro *observations, macrophage activation has been described as belonging to two main types, classically activated (or inflammatory) M1 and alternatively activated M2 macrophages. Each subpopulation is characterized by a distinct profile of gene expression and, accordingly, each has different functions [9,34]. M1 macrophages produce high levels of proinflammatory cytokines and mediators which are vital components of host defence [35]. M2 macrophages have rather an anti-inflammatory profile and are involved in tissue remodelling [36]. A recent *in vivo *study demonstrated that, in injured spinal cords, macrophages/microglia cells exhibit two phenotypes, while the M1 is neurotoxic, M2 promotes a regenerative growth response [37]. In the current *in vivo *study, we provide evidence that there is a distinctive phenotype of microglial activation in response to nerve lesion, inflammation and infection. Microglial activation following nerve lesion was characterized by morphologic changes and cell proliferation, but low levels of proinflammatory cytokine expression. In this, it resembles the M2 macrophage activation in peripheral tissue. In contrast, LPS-induced microglial activation possessed a typical inflammatory phenotype, compared to the one of peripheral M1 macrophages. Thus, the morphological and functional changes in microglia reflect altered activation states induced by different signals that arise from injured neurons or from circulating immune mediators. In addition to distinct phenotype of macrophage/microglia activation, our results also suggested that, indeed, the microglial cells may be a heterogeneous population, since monocytes in the blood have been characterized as inflammatory (CCR2^high^, CX3CR1^low ^and GR1^+^) and resident (CCR2^low ^CX3CR1 ^high ^GR1^-^) subpopulations [38]. Specific monocyte populations might give rise to specific tissue macrophages [39], including microglia.

### Potential contribution of CNS glial cells to pain in the different experimental conditions

In the last decades, considerable evidence was obtained supporting the concept that activated glial cells can contribute, in one way or another, to experimental pain states. More convincing data were accumulated in models of neuropathic pain following nerve lesions. The concept of glial involvement in pain modulation was initially developed following studies in which there was spinal glial activation [40], and was further supported by evidence of trigeminal glial activation in oro-facial pain conditions. Intracisternal administration of inhibitors for p38 or ERK1/2, MAPKs phosphorylated on activated glial cells, produced an anti-allodynic effect following infraorbital nerve injury [41]. Microglia inhibitor, minocycline, reduced the tactile hypersensitivity following transection of inferior alveolar nerve and mental nerve [42]. Studies applying the astrocyte inhibitor, fluoroacetate, demonstrated the important role of hyperactive trigeminal astrocytes in oro-facial neuropathic pain [43]. How these activated glial cells affect pain processing is always a hot topic and has not been fully answered. Proinflammatory cytokines have been suggested as signalling molecules between activated glial cells and their surrounding neurons to enhance pain transmission. In the absence of injury, central application of IL-1β and TNF-α induced allodynia and/or hyperalgesia [44,45]. Intrathecal administration of IL-1ra and soluble TNF-α [46,47] reduced enhanced nociceptive states. Unexpectedly, our results revealed that, in contrast with LPS induced microglial activation, mental nerve injury-induced CNS microglial activation had a specific phenotype with very low cytokine production profile, which is consistent with what we have observed previously in sciatic nerve injury-induced cytokine expression within the spinal cord [48]. Although such a low level of cytokine expression on highly activated microglia leads us to question the importance of glia-derived cytokines as a link in glia-to-neuron interactions and in the pathogenesis of neuropathic pain, we still cannot exclude the involvement of proinflammatory cytokines in this specific condition. Indeed, since cytokines act in autocrine and paracrine manner, they might need only to be produced in very low amounts to be functional. It is also possible that the level of proinflammatory cytokines is not enough to maintain hyperactivity of surrounding neurons, but might contribute to neuropathic pain though an indirect pathway, e.g., triggering functional changes of astrocytes and modulating the integrity of the blood-brain barrier. At the same time, it should be noticed that in the CNS microenvironment, proinflammatory cytokines are not exclusively secreted by activated glial cells. Indeed, damaged central primary afferent terminals have been shown to release these inflammatory molecules [49] and even blood born circulating cytokines can contribute to increase the local concentration in the microenvironment (unpublished personal data). In addition, growing evidence suggests that many other candidates expressed by activated microglia can contribute to modulate pain processing, such as cell surface receptors (P2X4, P2X7, CCR2, CX3CR1, etc) [50-54], enzymes [55] and complement components [56]. Evidence of CNS glial involvement in peripheral inflammatory pain is less substantial. Our results did not provide neither spatial nor temporal correlation between LPS- and CFA-induced CNS glial reaction and enhanced pain behaviour. However, some studies using other inflammatory stimuli, such as application of inflammatory irritant mustard oil in the tooth pulp, demonstrated that inhibition of P38 MAPK signaling and inhibition of astrocytes metabolic processes can completely abolish central sensitization in Sp5C nociceptive neurons [57,58].

In summary, this study identifies the distinctive phenotype of CNS glial cells in response to remote nerve injury and to local infection/inflammation, which all produced enhanced pain behaviour. It also specifies that both microglial and astrocytic activations are multi-dimensional. Functional and morphological changes were not time-locked, as one could be detected in the absence of the other, depending on the stimulus that triggered activation. Further functional studies will help to delineate whether and how CNS glial cells contribute to different pathological pain conditions.

## Competing interests

The authors declare that they have no competing interests.

## Authors' contributions

SHL carried out surgery, behavioral testing, immunohistochemistry and *in situ *hybridization experiments. YQZ was responsible for the experiments related to molecular biology. SHL also participated in data analysis and the preparation of the manuscript. AR contributed to the conception of the study and preparation of the manuscript. JZ conceived the project, lead the experimental design and data analysis and wrote the manuscript. All authors read and approved the final manuscript.
